# Publication of Study Exit Procedures in Clinical Trials of Deep Brain Stimulation: A Focused Literature Review

**DOI:** 10.3389/fnhum.2020.581090

**Published:** 2020-10-21

**Authors:** Lauren R. Sankary, Akila M. Nallapan, Olivia Hogue, Andre G. Machado, Paul J. Ford

**Affiliations:** ^1^Neuroethics Program, Cleveland Clinic, Cleveland, OH, United States; ^2^Neurological Institute, Cleveland Clinic, Cleveland, OH, United States; ^3^School of Graduate Studies, Case Western Reserve University, Cleveland, OH, United States; ^4^Department of Quantitative Health Sciences, Lerner Research Institute, Cleveland Clinic, Cleveland, OH, United States

**Keywords:** deep brain stimulation, neuromodulation, research ethics, neuroethics, review, neuro-psychiatric disorders

## Abstract

Considerable variability exists in the publication of clinical research study procedures related to study enrollment and participant exit from clinical trials. Despite recent efforts to encourage research data sharing and greater transparency regarding research outcomes, reporting of research procedures remains inconsistent. Transparency about study procedures has important implications for the interpretation of study outcomes and the consistent implementation of best practices in clinical trial design and conduct. This review of publications from clinical trials of deep brain stimulation (DBS) using the MEDLINE database examines the frequency and consistency of publication of research procedures and data related to exit from DBS research. Related considerations, such as device explant or continued use, battery and other device hardware replacements, and post-trial follow-up care are also reviewed. This review finds significant variability in the publication and reporting of study exit procedures. Of the 47 clinical trials included in this review, 19% (9) disclosed procedures related to exit from research. Reporting of other exit-related data and study procedures examined in this review was identified in fewer than half of the included clinical trials. The rate of participant retention and duration of follow-up was reported more than any other category of data included in this review. Results inform efforts to improve consistency in research design, conduct, and publication of results from clinical trials in DBS and related areas of clinical research.

## Introduction

Distinct ethical considerations arise as research participants exit clinical trials for investigational brain implants, such as deep brain stimulation (DBS) as compared to their initial entry into the study. Well beyond the initial informed consent process during study enrollment, research participants face complex decisions about the surgical removal or continued use of investigational implants upon exiting from research. The complexity of these decisions and variability in study exit procedures amplifies the importance of the transparent publication of data and study procedures in DBS research, particularly concerning participant withdrawal and study outcomes. Greater transparency regarding research procedures for exit from clinical research, retention rate, and reasons for attrition will serve to inform human subjects protection and enhance consistent implementation of best ethical practices in brain device research.

This focused literature review examines how frequently and consistently published results from clinical trials of DBS describe procedures for study exit and data related to the number of research participants who exit or withdraw from these studies. Related data on device explant or continued device use by participant, battery, and other device hardware replacements, and post-trial follow-up care are also reviewed. These findings should inform guidelines for data and study procedures disclosed in publications of findings in DBS research.

## Background

The transparent publication of research findings informs ongoing research efforts and supports evidence-based decisions in medicine. In addition to transparent reporting of research outcomes, transparency regarding study procedures carries important implications for the interpretation of research findings and the development of best practices in clinical trial design and conduct. A desire to reduce reporting bias, including the omission or concealment of adverse events and the exaggeration of reported efficacy, led to the establishment of clinical trials registries such as clinicaltrials.gov in 2000 and the WHO International Trials Registry Platform in 2005 (Joshi and Bhardwaj, 2018). Recent data transparency initiatives call for research data sharing and greater transparency in the publication of research procedures and outcomes (Zorzela et al., 2016; Ioannidis et al., 2017; Munafò et al., 2017). In the United States, updates to the Final Rule for Clinical Trials Registration and Results Information Submission [42 CFR 11.48(a; 5)] expand required disclosures to include submission of “a copy of the protocol and statistical analysis plan (if not included in the protocol), including all amendments” for clinical trials initiated on or after January 18, 2017. This new requirement holds promise to improve publicly available information about clinical trial procedures; however, compliance has been inconsistent across research domains (DeVito et al., 2020; Piller, 2020) and requirements under the final rule do not apply to an important subset of clinical trials, including device feasibility studies.

A lack of standardization of protocol elements has particularly important implications in DBS research in which each clinical trial may contain a relatively small number of research participants. Inconsistent reporting of study procedures limits understanding and replicability of research outcomes, leads to variable research practices in clinical trials of DBS, and constrains the development of best practices in clinical research in DBS and other implanted brain devices. Inconsistent research practices may also exacerbate disparities in access to research opportunities and investigational therapies.

However, there continue to be substantial hurdles to systematically reviewing and identifying inconsistencies in research practices. Deficiencies in reporting of data on adverse events, participant withdrawal, and long-term outcomes contribute to the risk of bias in systematic reviews (Zorzela et al., 2016). In 2015, a PRISMA harms checklist was developed to improve harms reporting in systematic reviews to promote a more balanced assessment of benefits and harms.

While publication of research results in DBS may vary according to journal specifications and manuscript requirements, guidelines such as the CONSORT checklist create some consistency across journals. The CONSORT flow diagram calls for the visual representation of the number of potential participants excluded from study enrollment, the number of participants who did not receive allocated interventions (with reasons), those lost to follow-up (with reasons), and those who discontinued the study intervention (with reasons). Of the 25-item checklist prescribed by the CONSORT 2010 Statement, at least four items relate to procedures for study termination and participant withdrawal: “participant losses and exclusions after randomization, together with reasons” (Item 13b), “dates defining periods of recruitment and follow-up” (Item 14a), “why the trial ended or was stopped” (Item 14b), and “where the full trial protocol can be accessed, if available” (Item 24). Recognizing the need to move the field forward concerning the reporting of data on and procedures for participant withdrawal or exit from research, the following literature review was undertaken.

## Focused Literature Review

A focused review of the published results from clinical trials of DBS was carried out using the MEDLINE database^1^. The authors selected studies with English-language published results in which DBS was the primary research intervention. The following search terms were used: DBS, clinical trials, trial registration number, National Clinical Trial (NCT). Trial identifiers, such as NCT numbers, were used to identify any other articles reporting data from the included trial and to access additional trial information^1^; Supplementary Material were accessed and reviewed when relevant.

### Data Extraction

Two reviewers (LS and AN) independently extracted data from published articles^1^. Two inter-rater disagreements regarding article inclusion and characterization of data were mediated by a third reviewer (PF). Extracted data elements include the publication of study protocol, description of exit procedures, participant retention rate, duration of follow-up, reasons disclosed for study exit or withdrawal, and data on device explant or hardware replacements.

### Study Selection and Trial Characteristics

A total of 2,906 titles and abstracts were screened, of which 143 full-length articles met the criteria for further review. Articles were included if they reported procedures or results from a clinical trial in human subjects for which DBS was the primary research intervention. Case reports and articles reporting observational research or basic science findings or reviewing surgical techniques were excluded from this review. After the removal of duplicates and articles that did not meet inclusion criteria, 71 articles published between 2007 and 2019 remained and are included in this review. These articles provided data from 47 unique clinical trials. For reporting purposes, this review considers a clinical trial to be the unit of observation. That is, if a data element of interest was included in any publication associated with a single trial, the item is coded as present in publication for that trial. The full PRISMA flow diagram, with reasons for article exclusions, can be found in Supplementary Figure 1.

Parkinson’s disease (*n* = 12) and major depression (*n* = 13) were the most common trial indications, followed by dystonia (*n* = 4) and Tourette syndrome (*n* = 3). The following were represented by two or fewer trials: addiction, Alzheimer’s disease, anorexia nervosa, chronic pain, cluster headache, essential tremor, Huntington’s disease, minimally conscious state, morbid obesity, multiple sclerosis, obsessive-compulsive disorder. Eighteen trials were conducted in the United States (27 international, 2 unknown). Nineteen trials were characterized as “pilot,” “feasibility,” “planning” or “Phase 1” trials; three were labeled “Phase 2” and the remainder did not specify a specific phase of research.

### Publication of Study Exit Procedures

Forty of the included trials were complete at the time of this review. Of these, the median retention rate at the final follow-up was 92% (range 44 to 100%, 37 of 40 complete trials reporting). Among the 45 trials that described the duration of follow-up, the median follow-up time was 12 months (range 1.5–84 months; includes actual follow-up times where available and reported and planned follow-up times otherwise).

**Figure 1:**
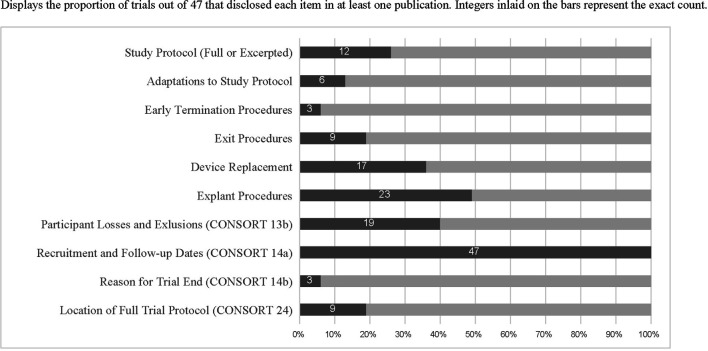
Displays the proportion of trials out of 47 that disclosed each item in at least one publication. Integers inlaid on the bars represent the exact count.

Table 1 displays examples of robust reporting of study exit-related information from articles included in this review. Figure 1 displays the counts and proportions of select reporting items of interest. Rates of reporting for each item were generally low, except for CONSORT 2010 item 14a (“dates defining periods of recruitment and follow-up”). Further details outside the scope of Figure 1 are described below.

**Table 1:** Examples of robust reporting of study exit-related information.

Description of study exit procedures
“7.14 Discontinuation/withdrawal of participants and “stopping rules”: Patients wishing to discontinue participation with the trial will be free to do so. The reasons for withdrawal will be sought from all individuals and recorded. Adverse events will be recorded systematically throughout the trial and from all patients wishing to withdraw. Appropriate medical advice and treatment will be made available to any individuals experiencing adverse events from trial participation. The trial will be stopped prematurely if there are doubts regarding the safety or scientific validity of its continuation, following the principles of Good Clinical Practice and the Medicines for Human Use (Clinical Trials) Regulations 2004 Part 4” (Gratwicke et al., 2018).
“At the end of the 15-month protocol period, the patient has the option to remove the DBS device which can be done in a simple operation with minimal risks. There will be an option for an annual research follow-up for up to 4 years. At the end of the protocol period, or at any point subsequently, the patient has the option to remove the DBS device which can be done in a simple operation with minimal risks. If the participant decides to keep the DBS stimulator *in situ*, they will have routine neurosurgical DBS follow-up every 12 months for clinical care” (Park et al., 2018, p. 6).
“Participants completing the 12-month study were invited to continue in a long-term, naturalistic follow-up study. Study visits occurred every 6 months. Changes in stimulation parameters, medications, and psychotherapy were allowed. For patients continuing with chronic DBS, a rechargeable battery was provided as needed” (Plow et al., 2013, p. 843).
**Reporting of data on study exit**
“One patient had his DBS system removed after 1 year due to the device becoming the object of his obsession. A second patient requested to have the device removed because it caused him severe distress, and had become a part of his obsession syndrome. He wanted to constantly feel the stimulation. The device was removed without complication 21 months after implantation. After explantation, the patient was lost to follow-up” (Lee et al., 2019, p. 34).
“Four participants withdrew consent before completing the open stimulation phase…. More than half (five of nine) of the participants responded positively. These five participants elected to continue DBS at the end of the trial, and their non-rechargeable implantable stimulator was replaced with a rechargeable stimulator for continued use. The remaining four of nine participants elected to have their DBS systems removed before completing the 18-month open stimulation phase” (Plow et al., 2013, p. 657).
**Reporting of hardware-related considerations**
“Three patients (2.4%) had complete removal of the device during the study: two due to infection, and one due to the patient’s choice. Four patients (3.1%) had leads repositioned to improve tremor control; one patient had repositioning at 3 months, one at 1 year, and two more than 2 years after implantation. Extension leads were replaced in seven patients (5.5%) due to malfunction (fracture or intermittent stimulation); three patients had the extension leads replaced at 6 months, one patient had it replaced after 15 months; one patient had it replaced after 18 months, two patients had extension lead replacement after more than 2 years. IPG malfunction occurred in three patients (2.4%), necessitating replacement earlier than expected” (Wharen et al., 2017, p. 23–24).

When procedures for study exit were described (*n* = 9 trials reporting this information), they were located in a published protocol 100% of the time. Nineteen trials reported both the number of research participants who exited the study and the reasons for attrition and three also published detailed study exit procedures. Only three trials reported both explant procedures and detailed exit procedures. Twenty-five trials reported retention rates of less than 100%. Among these, 24 reported numbers of exited research participants and 19 reported reasons for the exit, showing incomplete reporting of CONSORT item 13(b). Only four clinical trials specifically described ethical concerns related to study exit. Supplementary Table 1 includes all included trials and reporting items identified as present or absent for each.

Only three of the included articles were published before the 2010 publication date of the CONSORT guidelines. None of these included a description of exit procedures, number of exited research participants, nor reasons for exit.

## Discussion

Reported study procedures were insufficient to evaluate differences in research practices across clinical trials. Options related to device explant or continued use upon the termination of the study and the provision of post-trial follow-up care were difficult to discern from the published literature included in this review. Fewer than one-fifth (9) of the clinical trials with published research findings described procedures for participant withdrawal or exit upon study termination. Even fewer (6) of the clinical trials with published findings examined in this review described adaptations to study procedures or the study protocol. As DBS research continues to develop and flexible study designs are used more frequently, reporting of adaptations to study procedures or the study protocol may improve.

Reporting of outcomes and data on how many research participants exit from clinical trials was more substantial. However, the reviewed articles show inconsistent adherence to the CONSORT guidelines in reporting this data. CONSORT flow diagrams were frequently used to illustrate information about participant exclusion and retention. Emphasis was placed on representation of the number of potential participants excluded from study enrollment and the number of participants who did not receive allocated interventions (with reasons), rather than a comprehensive explanation of reasons participants were lost to follow-up or discontinued the study intervention. One potential explanation for this is the frequency with which study results are reported before a clinical trial has ended or the final follow-up visit has been completed. Additionally, numerical data may be reported more frequently than reasons for attrition due to the practical challenges of obtaining explanations from participants with whom the study team has lost contact. While it may be more feasible to obtain reasons for discontinuation of study intervention, data collection still may depend upon a participant’s willingness to share this reasoning after they have decided to exit from the study.

### Ethical Implications of Inconsistent Reporting

Gaps in publicly available information about study exit procedures may conceal variation in procedures related to DBS device removal and post-trial access to DBS devices. Arbitrary variability in exit procedures across clinical trials creates potential disparities in ongoing risks research participants may be exposed to as a result of participation and exit from research (including risks of infection, hardware malfunction, and surgical complications during explant). Similarly, variability in options provided for post-trial use of investigational implants creates disparities in direct benefit associated with research opportunities. In both examples, research participants who might be able to choose between entering different protocols do not have the assurance of uniform protections or the information to distinguish between procedural differences amongst alternatives. These justice considerations suggest an ethical imperative to improve reporting of study exit procedures.

Data on the number of participants who withdraw from a clinical trial may be more ethically complex to report. Concern to protect the confidentiality and to respect a research participant’s decision to withdraw from research may limit the amount of data researchers can obtain regarding reasons for participant-initiated withdrawal. However, it is important to disclose criteria investigators use for exclusion or termination of a participant’s ongoing participation in a clinical trial. This should always be undertaken in a way that is value-neutral and does not cast blame on research participants.

Transparent reporting of research findings in DBS can enhance the generalizability of knowledge gained from the contributions of research participants. Informed consent to enrollment in a clinical trial of DBS will be enhanced by promoting public availability of information about study procedures and outcomes. Finally, reporting of procedures related to study exit serves to inform research oversight and support the development of evidence-based best practices in DBS research. As investigations into new applications of DBS continue, it is vitally important to enhance consistency in the publication of both study procedures and outcomes from clinical trials of DBS. We encourage DBS researchers to disclose the following data and study procedures consistently to maximize transparency in the publication of DBS research findings concerning those participants who exit the research study: criteria for treatment response/non-response; adaptations to the study protocol, including early termination of the study; data on participant-initiated and investigator-initiated withdrawal from research participation, including reasons; battery and device hardware replacements; options provided related to device explant or continued use (including provisions for post-trial follow-up care and device hardware replacements); and duration of follow-up contact with each study participant. Additional guidelines for the transparent publication of research procedures and findings in DBS and other areas of clinical research are needed. The authors hope the proposed list can serve as a first step in facilitating more consistent reporting practices across clinical trials of DBS and other investigational brain implants.

## Author Contributions

LS designed the project, performed data collection, data analysis, and led the writing of this manuscript. AN contributed to data collection, data analysis, and the preparation of this manuscript. OH, AM, and PF contributed to data analysis and the preparation of this manuscript. All authors contributed to the article and approved the submitted version.

## Conflict of Interest

LS reports funding from National Institutes of Health (NIH) grant number F32MH115419 during the conduct of the study. AM reports distribution rights from Enspire DBS, personal fees from St Jude, grants from St Jude, grants from Medtronic, and grants from NIH, outside the submitted work. The remaining authors declare that the research was conducted in the absence of any commercial or financial relationships that could be construed as a potential conflict of interest.

## Footnotes

^1^https://clinicaltrials.gov/
