# Supplementary material for: Publication of Study Exit Procedures in Clinical Trials of Deep Brain Stimulation: A Focused Literature Review
Source: Front Hum Neurosci. 2020 Oct 21;14:581090. doi: 10.3389/fnhum.2020.581090 (PMC7609884; doi:10.3389/fnhum.2020.581090)
Supplement: Supplementary file 1 [file Data_Sheet_1.docx]

Supplementary Material

# Supplementary Figures

# Supplementary Figure 1. PRISMA Flow Diagram for Focused Literature Review

#
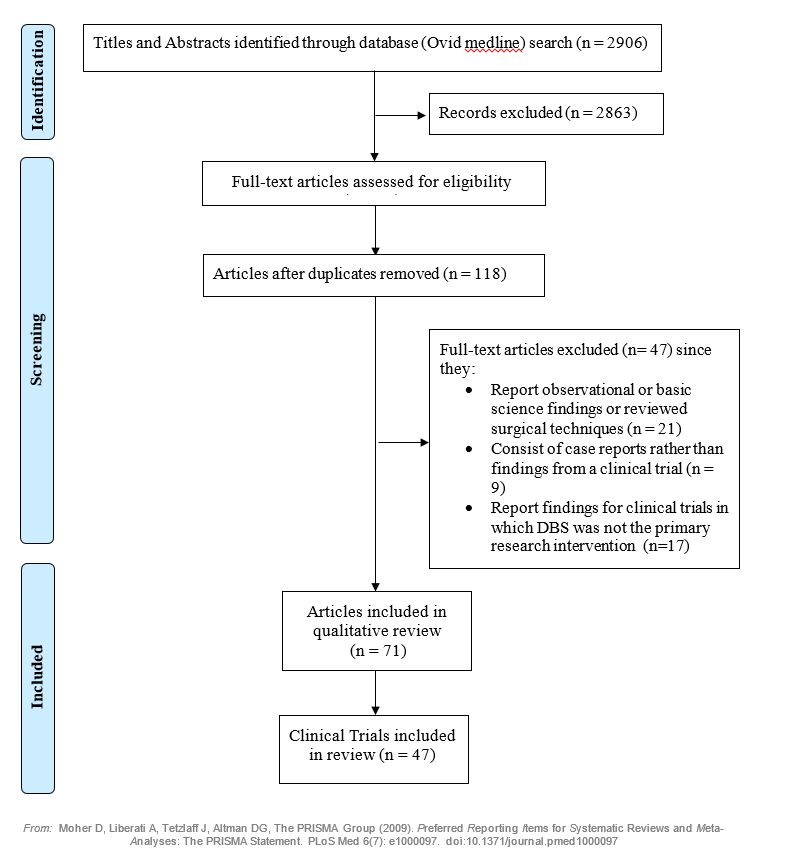


**Supplementary Table 1.** Exit procedures and data reported in all included trials (identified as present (X) or absent for each item)

| **#** | **TRIAL IDENTIFIER** | **PUBLICATIONS** | **PHASE** | **PROTOCOL** | **EXIT PROCEDURES** | **RETENTION RATE** | **EXIT DATA** | **REASONS FOR EXIT** | **EXPLANT** | **DEVICE REPLACEMENT** | **FOLLOW-UP DURATION** | **EARLY TERMINATION** | **PROTOCOL ADAPTATIONS** |
| --- | --- | --- | --- | --- | --- | --- | --- | --- | --- | --- | --- | --- | --- |
| 1 | NCT02549859 | 1 |  |  |  | X |  |  | X |  | X |  |  |
| 2 | NCT01701544 | 1 | X | X | X | X |  |  | X |  | X | X |  |
| 3 | NCT01924598 | 3 |  | X | X |  |  |  |  |  | X |  |  |
| 4 | NCT02288468 | 2 | X | X | X |  |  |  |  |  | X | X |  |
| 5 | NCT00617162 | 1 |  |  | X | X | X | X | X | X | X | X |  |
| 6 | NCT01922388 | 2 |  | X |  |  |  |  |  |  | X |  |  |
| 7 | NCT00282152 | 3 | X |  |  | X | X | X |  |  | X |  |  |
| 8 | NCT01476540 | 2 | X |  |  | X | X | X | X | X | X |  |  |
| 9 | NCT01703598 | 1 |  |  |  | X |  |  | X |  | X |  |  |
| 10 | NCT00954421 | 1 |  |  |  | X | X |  | X | X | X |  | X |
| 11 | NCT00478842 | 1 |  |  |  | X | X | X | X | X | X |  |  |
| 12 | NCT00773604 | 1 | X |  |  | X | X | X | X |  | X |  |  |
| 13 | NCT00837486 | 1 |  |  |  | X | X | X | X | X | X |  |  |
| 14 | NCT02491554 | 1 | X | X |  |  |  |  | X |  | X |  |  |
| 15 | NCT01027572 | 1 |  |  |  | X | X | X | X |  | X |  |  |
| 16 | NCT01608061 | 2 | X |  |  |  |  |  | X | X | X |  |  |
| 17 | NCT02046330 | 2 | X | X (PET) |  |  | X | X |  |  | X |  | X |
| 18 | NCT00792532 | 2 |  |  |  | X | X | X | X | X | X |  |  |
| 19 | NCT00367003 | 3 |  |  |  | X | X |  | X | X | X |  | X |
| 20 | NCT00148889 | 2 |  |  |  | X | X | X | X |  | X |  |  |
| 21 | NCT02235792 | 1 |  |  |  | X |  |  |  |  | X |  |  |
| 22 | NCT01221948 | 1 |  |  |  | X | X | X |  |  | X |  |  |
| 23 | NCT01268137 | 2 |  |  |  | X | X | X |  |  | X |  |  |
| 24 | NCT00056563 and NCT01076452 | 4 |  |  |  | X | X |  |  |  | X |  |  |
| 25 | NCT00902889 | 1 | X |  |  | X |  |  |  |  | X |  |  |
| 26 | NCT01569711 | 1 | X |  |  | X | X | X |  |  | X |  |  |
| 27 | NCT01072656 | 2 | X | X | X | X | X |  | X | X | X |  |  |
| 28 | NCT01095263 | 3 | X |  |  | X | X |  | X |  | X |  | X |
| 29 | NCT01329198 | 1 | X |  |  | X |  |  |  |  | X |  |  |
| 30 | NCT00142259 | 2 |  |  |  | X |  |  | X | X | X |  |  |
| 31 | NCT00552474 | 1 |  |  |  | X | X | X |  |  | X |  |  |
| 32 | NCT01355835 | 1 | X | X | X |  |  |  |  |  | X |  |  |
| 33 | NCT00122031 | 1 |  |  |  | X |  |  |  |  | X |  |  |
| 34 | NCT00057603 | 2 | X | X | X | X |  |  |  | X | X |  |  |
| 35 | NCT00662935 | 1 |  |  |  | X |  |  | X | X | X |  | X |
| 36 | NCT00139308 | 1 | X |  |  | X |  |  |  |  | X |  |  |
| 37 | NCT00132990 | 1 | X |  |  | X |  |  |  |  | X |  |  |
| 38 | NCT00196911 | 1 |  |  |  | X | X | X |  |  | X |  |  |
| 39 | NCT02087046 | 2 |  |  |  | X | X | X | X | X | X |  | X |
| 40 | NCT01778790 | 1 | X |  |  | X | X | X | X | X | X |  |  |
| 41 | NCT03425616 | 1 |  | X |  |  |  |  |  |  | X |  |  |
| 42 | NCT02232919 | 1 | X | X | X |  |  |  |  |  | X |  |  |
| 43 | NCT01559220 | 1 | X |  |  | X |  |  |  |  | X |  |  |
| 44 | Unregistered: Raymakers et al. 2017 | 1 |  |  |  |  |  |  |  |  | X |  |  |
| 45 | Unregistered: Lee et al. 2019 Phase 1 Pilot | 1 | X |  |  | X | X | X | X | X | X |  |  |
| 46 | ACTRN12611000889954 | 1 | X |  |  | X |  |  | X | X | X |  |  |
| 47 | NTR2118 | 2 |  | X | X | X | X | X |  | X | X |  |  |

**Supplementary Table 2.** Full-text articles included in qualitative review.

1. Bergfeld, I. O., Mantione, M., Hoogendoorn, M. L. C., Ruhé, H. G., Notten, P., van Laarhoven, J., et al. (2016). Deep Brain Stimulation of the Ventral Anterior Limb of the Internal Capsule for Treatment-Resistant Depression: A Randomized Clinical Trial. *JAMA Psychiatry* 73, 456-465. doi:[10.1001/jamapsychiatry.2016.0152](https://doi.org/10.1001/jamapsychiatry.2016.0152).
2. Bewernick, B. H., Kayser, S., Gippert, S. M., Switala, C., Coenen, V. A., and Schlaepfer, T. E. (2017). Deep brain stimulation to the medial forebrain bundle for depression- long-term outcomes and a novel data analysis strategy. *Brain Stimulation* 10, 664–671. doi:[10.1016/j.brs.2017.01.581](https://doi.org/10.1016/j.brs.2017.01.581).
3. Brodsky, M. A., Anderson, S., Murchison, C., Seier, M., Wilhelm, J., Vederman, A., et al. (2017). Clinical outcomes of asleep vs awake deep brain stimulation for Parkinson disease. *Neurology* 89, 1944–1950. doi:[10.1212/WNL.0000000000004630](https://doi.org/10.1212/WNL.0000000000004630).
4. Charles, P. D., Dolhun, R. M., Gill, C. E., Davis, T. L., Bliton, M. J., Tramontana, M. G., et al. (2012). Deep brain stimulation in early Parkinson’s disease: Enrollment experience from a pilot trial. *Parkinsonism & Related Disorders* 18, 268–273. doi:[10.1016/j.parkreldis.2011.11.001](https://doi.org/10.1016/j.parkreldis.2011.11.001).
5. Coenen, V. A., Bewernick, B. H., Kayser, S., Kilian, H., Boström, J., Greschus, S., et al. (2019). Superolateral medial forebrain bundle deep brain stimulation in major depression: a gateway trial. *Neuropsychopharmacol.* 44, 1224–1232. doi:[10.1038/s41386-019-0369-9](https://doi.org/10.1038/s41386-019-0369-9).
6. Coenen, V. A., Rijntjes, M., Prokop, T., Piroth, T., Amtage, F., Urbach, H., et al. (2016). One-pass deep brain stimulation of dentato-rubro-thalamic tract and subthalamic nucleus for tremor-dominant or equivalent type Parkinson’s disease. *Acta Neurochir* 158, 773–781. doi:[10.1007/s00701-016-2725-4](https://doi.org/10.1007/s00701-016-2725-4).
7. Coenen, V. A., Sajonz, B., Reisert, M., Bostroem, J., Bewernick, B., Urbach, H., et al. (2018). Tractography-assisted deep brain stimulation of the superolateral branch of the medial forebrain bundle (slMFB DBS) in major depression. *NeuroImage: Clinical* 20, 580–593. doi:[10.1016/j.nicl.2018.08.020](https://doi.org/10.1016/j.nicl.2018.08.020).
8. De Salles, A. A. F., Barbosa, D. A. N., Fernandes, F., Abucham, J., Nazato, D. M., Oliveira, J. D., et al. (2018). An Open-Label Clinical Trial of Hypothalamic Deep Brain Stimulation for Human Morbid Obesity: BLESS Study Protocol. *Neurosurgery* 83, 800–809. doi:[10.1093/neuros/nyy024](https://doi.org/10.1093/neuros/nyy024).
9. Derrey, S., Chastan, N., Maltete, D., Verin, E., Dechelotte, P., Lefaucheur, R., et al. (2015). Impact of deep brain stimulation on pharyngo-esophageal motility: a randomized cross-over study. *Neurogastroenterol. Motil.* 27, 1214–1222. doi:[10.1111/nmo.12607](https://doi.org/10.1111/nmo.12607).
10. Deuschl, G., Schade-Brittinger, C., Krack, P., Volkmann, J., Schäfer, H., Bötzel, K., et al. (2006). A randomized trial of deep-brain stimulation for Parkinson’s disease. *N. Engl. J. Med.* 355, 896–908. doi:[10.1056/NEJMoa060281](https://doi.org/10.1056/NEJMoa060281).
11. Dinkelbach, L., Mueller, J., Poewe, W., Delazer, M., Elben, S., Wolters, A., et al. (2015). Cognitive outcome of pallidal deep brain stimulation for primary cervical dystonia: One year follow up results of a prospective multicenter trial. *Parkinsonism & Related Disorders* 21, 976–980. doi:[10.1016/j.parkreldis.2015.06.002](https://doi.org/10.1016/j.parkreldis.2015.06.002).
12. Fenoy, A. J., Schulz, P. E., Selvaraj, S., Burrows, C. L., Zunta-Soares, G., Durkin, K., et al. (2018). A longitudinal study on deep brain stimulation of the medial forebrain bundle for treatment-resistant depression. *Transl Psychiatry* 8, 111. doi:[10.1038/s41398-018-0160-4](https://doi.org/10.1038/s41398-018-0160-4).
13. Fenoy, A. J., Schulz, P., Selvaraj, S., Burrows, C., Spiker, D., Cao, B., et al. (2016). Deep brain stimulation of the medial forebrain bundle: Distinctive responses in resistant depression. *Journal of Affective Disorders* 203, 143–151. doi:[10.1016/j.jad.2016.05.064](https://doi.org/10.1016/j.jad.2016.05.064).
14. Filkowski, M. M., Mayberg, H. S., and Holtzheimer, P. E. (2016). Considering Eligibility for Studies of Deep Brain Stimulation for Treatment-Resistant Depression: Insights From a Clinical Trial in Unipolar and Bipolar Depression. *The Journal of ECT* 32, 122–126. doi:[10.1097/YCT.0000000000000281](https://doi.org/10.1097/YCT.0000000000000281).
15. Fitzgerald, P. B., Segrave, R., Richardson, K. E., Knox, L. A., Herring, S., Daskalakis, Z. J., et al. (2018). A pilot study of bed nucleus of the stria terminalis deep brain stimulation in treatment-resistant depression. *Brain Stimulation* 11, 921–928. doi:[10.1016/j.brs.2018.04.013](https://doi.org/10.1016/j.brs.2018.04.013).
16. Follett, K. A., Hur, K., Marks, W. J., Moy, C., Hogarth, P., Holloway, K., et al. (2010). Pallidal versus Subthalamic Deep-Brain Stimulation for Parkinson’s Disease. *N. Eng. J. Med.* 362, 2077-2091. doi: 10.1056/NEJMoa0907083.
17. Fontaine, D., Lazorthes, Y., Mertens, P., Blond, S., Géraud, G., Fabre, N., et al. (2010). Safety and efficacy of deep brain stimulation in refractory cluster headache: a randomized placebo-controlled double-blind trial followed by a 1-year open extension. *J Headache Pain* 11, 23–31. doi:[10.1007/s10194-009-0169-4](https://doi.org/10.1007/s10194-009-0169-4).
18. Goodman, W. K., Foote, K. D., Greenberg, B. D., Ricciuti, N., Bauer, R., Ward, H., et al. (2010). Deep Brain Stimulation for Intractable Obsessive Compulsive Disorder: Pilot Study Using a Blinded, Staggered-Onset Design. *Biological Psychiatry* 67, 535–542. doi:[10.1016/j.biopsych.2009.11.028](https://doi.org/10.1016/j.biopsych.2009.11.028).
19. Gratwicke, J., Zrinzo, L., Kahan, J., Peters, A., Beigi, M., Akram, H., et al. (2018). Bilateral Deep Brain Stimulation of the Nucleus Basalis of Meynert for Parkinson Disease Dementia: A Randomized Clinical Trial. *JAMA Neurol.* 75, 169-178. doi:[10.1001/jamaneurol.2017.3762](https://doi.org/10.1001/jamaneurol.2017.3762).
20. Grubert, C., Hurlemann, R., Bewernick, B. H., Kayser, S., Hadrysiewicz, B., Axmacher, N., et al. (2011). Neuropsychological safety of nucleus accumbens deep brain stimulation for major depression: Effects of 12-month stimulation. *The World Journal of Biological Psychiatry* 12, 516–527. doi:[10.3109/15622975.2011.583940](https://doi.org/10.3109/15622975.2011.583940).
21. Hacker, M. L., DeLong, M. R., Turchan, M., Heusinkveld, L. E., Ostrem, J. L., Molinari, A. L., et al. (2018). Effects of deep brain stimulation on rest tremor progression in early stage Parkinson disease. *Neurology* 91, e463–e471. doi:[10.1212/WNL.0000000000005903](https://doi.org/10.1212/WNL.0000000000005903).
22. Holtzheimer, P. E. (2012). Subcallosal Cingulate Deep Brain Stimulation for Treatment-Resistant Unipolar and Bipolar Depression. *Arch Gen Psychiatry* 69, 150-158. doi:[10.1001/archgenpsychiatry.2011.1456](https://doi.org/10.1001/archgenpsychiatry.2011.1456).
23. Holtzheimer, P. E., Husain, M. M., Lisanby, S. H., Taylor, S. F., Whitworth, L. A., McClintock, S., et al. (2017). Subcallosal cingulate deep brain stimulation for treatment-resistant depression: a multisite, randomised, sham-controlled trial. *The Lancet Psychiatry* 4, 839–849. doi:[10.1016/S2215-0366(17)30371-1](https://doi.org/10.1016/S2215-0366(17)30371-1).
24. Jiang, L., Poon, W. S., Moro, E., Xian, W., Yang, C., Zhu, X. L., et al. (2017). Early versus Late Application of Subthalamic deep brain Stimulation to Parkinson’s disease patients with motor complications (ELASS): protocol of a multicentre, prospective and observational study. *Open Access* 7:e018610. doi: 10.1136/bmjopen-2017-018610
25. Kiss, Z. H. T., Doig-Beyaert, K., Eliasziw, M., Tsui, J., Haffenden, A., and Suchowersky, O. (2007). The Canadian multicentre study of deep brain stimulation for cervical dystonia. *Brain* 130, 2879–2886. doi:[10.1093/brain/awm229](https://doi.org/10.1093/brain/awm229).
26. Kubu, C. S., Brelje, T., Butters, M. A., Deckersbach, T., Malloy, P., Moberg, P., et al. (2017). Cognitive outcome after ventral capsule/ventral striatum stimulation for treatment-resistant major depression. *J Neurol Neurosurg Psychiatry* 88, 262–265. doi:[10.1136/jnnp-2016-313803](https://doi.org/10.1136/jnnp-2016-313803).
27. Kupsch, A., Trottenberg, T., Eisner, W., Deuschl, G., Roeste, G. K., Krause, M., et al. (2006). Pallidal deep-brain stimulation in primary generalized or segmental dystonia*. N. Engl. J. Med*. 355, 1978–1990. doi: 10.1056/NEJMoa063618
28. Lee, D. J., Dallapiazza, R. F., De Vloo, P., Elias, G. J. B., Fomenko, A., Boutet, A., et al. (2019). Inferior thalamic peduncle deep brain stimulation for treatment-refractory obsessive-compulsive disorder: A phase 1 pilot trial. *Brain Stimulation* 12, 344–352. doi:[10.1016/j.brs.2018.11.012](https://doi.org/10.1016/j.brs.2018.11.012).
29. Lempka, S. F., Malone, D. A., Hu, B., Baker, K. B., Wyant, A., Ozinga, J. G., et al. (2017). Randomized clinical trial of deep brain stimulation for poststroke pain: DBS for Pain. *Ann Neurol.* 81, 653–663. doi:[10.1002/ana.24927](https://doi.org/10.1002/ana.24927).
30. Lipsman, N., Lam, E., Volpini, M., Sutandar, K., Twose, R., Giacobbe, P., et al. (2017). Deep brain stimulation of the subcallosal cingulate for treatment-refractory anorexia nervosa: 1 year follow-up of an open-label trial. *The Lancet Psychiatry* 4, 285–294. doi:[10.1016/S2215-0366(17)30076-7](https://doi.org/10.1016/S2215-0366(17)30076-7).
31. Lipsman, N., Woodside, D. B., Giacobbe, P., Hamani, C., Carter, J. C., Norwood, S. J., et al. (2013). Subcallosal cingulate deep brain stimulation for treatment-refractory anorexia nervosa: a phase 1 pilot trial. *The Lancet* 381, 1361–1370. doi:[10.1016/S0140-6736(12)62188-6](https://doi.org/10.1016/S0140-6736(12)62188-6).
32. Lozano, A. M., Fosdick, L., Chakravarty, M. M., Leoutsakos, J.-M., Munro, C., Oh, E., et al. (2016). A Phase II Study of Fornix Deep Brain Stimulation in Mild Alzheimer’s Disease. *J. Alzheimers Dis* 54, 777–787. doi:[10.3233/JAD-160017](https://doi.org/10.3233/JAD-160017).
33. Millan, S. H., Hacker, M. L., Turchan, M., Molinari, A. L., Currie, A. D., and Charles, D. (2017). Subthalamic Nucleus Deep Brain Stimulation in Early Stage Parkinson’s Disease Is Not Associated with Increased Body Mass Index. *Parkinsons Dis.* 2017, 7163801. doi:[10.1155/2017/7163801](https://doi.org/10.1155/2017/7163801).
34. Millet, B., Jaafari, N., Polosan, M., Baup, N., Giordana, B., Haegelen, C., et al. (2014). Limbic versus cognitive target for deep brain stimulation in treatment-resistant depression: Accumbens more promising than caudate. *European Neuropsychopharmacology* 24, 1229–1239. doi:[10.1016/j.euroneuro.2014.05.006](https://doi.org/10.1016/j.euroneuro.2014.05.006).
35. Mitchell, K. T., Larson, P., Starr, P. A., Okun, M. S., Wharen, R. E., Uitti, R. J., et al. (2019). Benefits and risks of unilateral and bilateral ventral intermediate nucleus deep brain stimulation for axial essential tremor symptoms. *Parkinsonism & Related Disorders* 60, 126–132. doi:[10.1016/j.parkreldis.2018.09.004](https://doi.org/10.1016/j.parkreldis.2018.09.004).
36. Okun, M. S., Foote, K. D., Wu, S. S., Ward, H. E., Bowers, D., Rodriguez, R. L., et al. (2013). A Trial of Scheduled Deep Brain Stimulation for Tourette Syndrome: Moving Away From Continuous Deep Brain Stimulation Paradigms. *JAMA Neurol* 70, 85-94. doi:[10.1001/jamaneurol.2013.580](https://doi.org/10.1001/jamaneurol.2013.580).
37. Okun, M. S., Gallo, B. V., Mandybur, G., Jagid, J., Foote, K. D., Revilla, F. J., et al. (2012). Subthalamic deep brain stimulation with a constant-current device in Parkinson’s disease: an open-label randomised controlled trial. *The Lancet Neurology* 11, 140–149. doi:[10.1016/S1474-4422(11)70308-8](https://doi.org/10.1016/S1474-4422(11)70308-8).
38. Oliveria, S. F., Rodriguez, R. L., Bowers, D., Kantor, D., Hilliard, J. D., Monari, E. H., et al. (2017). Safety and efficacy of dual-lead thalamic deep brain stimulation for patients with treatment-refractory multiple sclerosis tremor: a single-centre, randomised, single-blind, pilot trial. *The Lancet Neurology* 16, 691–700. doi:[10.1016/S1474-4422(17)30166-7](https://doi.org/10.1016/S1474-4422(17)30166-7).
39. Ostrem, J. L., San Luciano, M., Dodenhoff, K. A., Ziman, N., Markun, L. C., Racine, C. A., et al. (2017). Subthalamic nucleus deep brain stimulation in isolated dystonia: A 3-year follow-up study. *Neurology* 88, 25–35. doi:[10.1212/WNL.0000000000003451](https://doi.org/10.1212/WNL.0000000000003451).
40. Ostrem, J. L., Ziman, N., Galifianakis, N. B., Starr, P. A., Luciano, M. S., Katz, M., et al. (2016). Clinical outcomes using ClearPoint interventional MRI for deep brain stimulation lead placement in Parkinson’s disease. *JNS* 124, 908–916. doi:[10.3171/2015.4.JNS15173](https://doi.org/10.3171/2015.4.JNS15173).
41. Park, R. J., Scaife, J. C., and Aziz, T. Z. (2018). Study Protocol: Using Deep-Brain Stimulation, Multimodal Neuroimaging and Neuroethics to Understand and Treat Severe Enduring Anorexia Nervosa. *Front. Psychiatry* 9:24. doi:[10.3389/fpsyt.2018.00024](https://doi.org/10.3389/fpsyt.2018.00024).
42. Park, R. J., Singh, I., Pike, A. C., and Tan, J. O. A. (2017). Deep Brain Stimulation in Anorexia Nervosa: Hope for the Hopeless or Exploitation of the Vulnerable? The Oxford Neuroethics Gold Standard Framework. *Front. Psychiatry* 8:44. doi:[10.3389/fpsyt.2017.00044](https://doi.org/10.3389/fpsyt.2017.00044).
43. Plow, E. B., Malone, D. A., and Machado, A. (2013). Deep brain stimulation of the ventral striatum/anterior limb of the internal capsule in thalamic pain syndrome: study protocol for a pilot randomized controlled trial. *Trials* 14, 241. doi:[10.1186/1745-6215-14-241](https://doi.org/10.1186/1745-6215-14-241).
44. Ponce, F. A., Asaad, W. F., Foote, K. D., Anderson, W. S., Rees Cosgrove, G., Baltuch, G. H., et al. (2016). Bilateral deep brain stimulation of the fornix for Alzheimer’s disease: surgical safety in the ADvance trial. *J. Neurosurg.* 125, 75–84. doi:[10.3171/2015.6.JNS15716](https://doi.org/10.3171/2015.6.JNS15716).
45. Puigdemont, D., Pérez-Egea, R., Portella, M. J., Molet, J., de Diego-Adeliño, J., Gironell, A., et al. (2012). Deep brain stimulation of the subcallosal cingulate gyrus: further evidence in treatment-resistant major depression. *Int. J. Neuropsychopharm.* 15, 121–133. doi:[10.1017/S1461145711001088](https://doi.org/10.1017/S1461145711001088).
46. Puigdemont, D., Portella, M., Pérez-Egea, R., Molet, J., Gironell, A., Diego-Adeliño, J. de, et al. (2015). A randomized double-blind crossover trial of deep brain stimulation of the subcallosal cingulate gyrus in patients with treatment-resistant depression: a pilot study of relapse prevention. *J Psychiatry Neurosci* 40, 224–231. doi:[10.1503/jpn.130295](https://doi.org/10.1503/jpn.130295).
47. Qu, L., Ge, S., Li, N., Wang, W., Yang, K., Wu, P., et al. (2019). Clinical evaluation of deep brain stimulation of nucleus accumbens/anterior limb of internal capsule for opioid relapse prevention: protocol of a multicentre, prospective and double-blinded study. *BMJ Open* 9, e023516. doi:[10.1136/bmjopen-2018-023516](https://doi.org/10.1136/bmjopen-2018-023516).
48. Raymaekers, S., Luyten, L., Bervoets, C., Gabriëls, L., and Nuttin, B. (2017). Deep brain stimulation for treatment-resistant major depressive disorder: a comparison of two targets and long-term follow-up. *Transl Psychiatry* 7:e1251. doi:[10.1038/tp.2017.66](https://doi.org/10.1038/tp.2017.66).
49. Reinacher, P. C., Amtage, F., Rijntj.es, M., Piroth, T., Prokop, T., Jenkner, C., et al. (2018). One Pass Thalamic and Subthalamic Stimulation for Patients with Tremor-Dominant Idiopathic Parkinson Syndrome (OPINION): Protocol for a Randomized, Active-Controlled, Double-Blinded Pilot Trial. *JMIR Res Protoc* 7:e36. doi:[10.2196/resprot.8341](https://doi.org/10.2196/resprot.8341).
50. Riva-Posse, P., Holtzheimer, P. E., Garlow, S. J., and Mayberg, H. S. (2013). Practical Considerations in the Development and Refinement of Subcallosal Cingulate White Matter Deep Brain Stimulation for Treatment-Resistant Depression. *World Neurosurgery* 80, S27.e25-S27.e34. doi:[10.1016/j.wneu.2012.11.074](https://doi.org/10.1016/j.wneu.2012.11.074).
51. Rothlind, J. C., York, M. K., Carlson, K., Luo, P., Marks, W. J., Weaver, F. M., et al. (2015). Neuropsychological changes following deep brain stimulation surgery for Parkinson’s disease: comparisons of treatment at pallidal and subthalamic targets versus best medical therapy. *J Neurol Neurosurg Psychiatry* 86, 622–629. doi:[10.1136/jnnp-2014-308119](https://doi.org/10.1136/jnnp-2014-308119).
52. Sajonz, B. E. A., Amtage, F., Reinacher, P. C., Jenkner, C., Piroth, T., Kätzler, J., et al. (2016). Deep Brain Stimulation for Tremor Tractographic Versus Traditional (DISTINCT): Study Protocol of a Randomized Controlled Feasibility Trial. *JMIR Res Protoc* 5, e244. doi:[10.2196/resprot.6885](https://doi.org/10.2196/resprot.6885).
53. Scharre, D. W., Weichart, E., Nielson, D., Zhang, J., Agrawal, P., et al. (2018). Deep Brain Stimulation of Frontal Lobe Networks to Treat Alzheimer’s Disease. *J. Alzheimers Dis.* 62, 621–633. doi:[10.3233/JAD-170082](https://doi.org/10.3233/JAD-170082).
54. Schlaepfer, T. E., Bewernick, B. H., Kayser, S., Mädler, B., and Coenen, V. A. (2013). Rapid Effects of Deep Brain Stimulation for Treatment-Resistant Major Depression. *Biological Psychiatry* 73, 1204–1212. doi:[10.1016/j.biopsych.2013.01.034](https://doi.org/10.1016/j.biopsych.2013.01.034).
55. Starr, P. A., Markun, L. C., Larson, P. S., Volz, M. M., Martin, A. J., and Ostrem, J. L. (2014). Interventional MRI–guided deep brain stimulation in pediatric dystonia: first experience with the ClearPoint system: Clinical article. *PED* 14, 400–408. doi:[10.3171/2014.6.PEDS13605](https://doi.org/10.3171/2014.6.PEDS13605).
56. Timmermann, L., Jain, R., Chen, L., Maarouf, M., Barbe, M. T., Allert, N., et al. (2015). Multiple-source current steering in subthalamic nucleus deep brain stimulation for Parkinson’s disease (the VANTAGE study): a non-randomised, prospective, multicentre, open-label study. *The Lancet Neurology* 14, 693–701. doi:[10.1016/S1474-4422(15)00087-3](https://doi.org/10.1016/S1474-4422(15)00087-3).
57. van der Wal, J. M., Bergfeld, I. O., Lok, A., Mantione, M., Figee, M., Notten, P., et al. (2020). Long-term deep brain stimulation of the ventral anterior limb of the internal capsule for treatment-resistant depression. *J Neurol Neurosurg Psychiatry* 91, 189–195. doi:[10.1136/jnnp-2019-321758](https://doi.org/10.1136/jnnp-2019-321758).
58. van Horne, C. G., Quintero, J. E., Gurwell, J. A., Wagner, R. P., Slevin, J. T., and Gerhardt, G. A. (2017). Implantation of autologous peripheral nerve grafts into the substantia nigra of subjects with idiopathic Parkinson’s disease treated with bilateral STN DBS: a report of safety and feasibility. *J. Neurosurg* 126, 1140–1147. doi:[10.3171/2016.2.JNS151988](https://doi.org/10.3171/2016.2.JNS151988).
59. van Horne, C. G., Quintero, J. E., Slevin, J. T., Anderson-Mooney, A., Gurwell, J. A., Welleford, A. S., et al. (2018). Peripheral nerve grafts implanted into the substantia nigra in patients with Parkinson’s disease during deep brain stimulation surgery: 1-year follow-up study of safety, feasibility, and clinical outcome. *Journal of Neurosurgery* 129, 1550–1561. doi:[10.3171/2017.8.JNS163222](https://doi.org/10.3171/2017.8.JNS163222).
60. Volkmann, J., Mueller, J., Deuschl, G., Kühn, A. A., Krauss, J. K., Poewe, W., et al. (2014). Pallidal neurostimulation in patients with medication-refractory cervical dystonia: a randomised, sham-controlled trial. *The Lancet Neurology* 13, 875–884. doi:[10.1016/S1474-4422(14)70143-7](https://doi.org/10.1016/S1474-4422(14)70143-7).
61. Volkmann, J., Wolters, A., Kupsch, A., Müller, J., Kühn, A. A., Schneider, G.-H., et al. (2012). Pallidal deep brain stimulation in patients with primary generalised or segmental dystonia: 5-year follow-up of a randomised trial. *The Lancet Neurology* 11, 1029–1038. doi:[10.1016/S1474-4422(12)70257-0](https://doi.org/10.1016/S1474-4422(12)70257-0).
62. Vora, A. K., Ward, H., Foote, K. D., Goodman, W. K., and Okun, M. S. (2012). Rebound symptoms following battery depletion in the NIH OCD DBS cohort: Clinical and reimbursement issues. *Brain Stimulation* 5, 599–604. doi:[10.1016/j.brs.2011.10.004](https://doi.org/10.1016/j.brs.2011.10.004).
63. Waring, R. (2006). A Randomized Trial of Deep-Brain Stimulation for Parkinson’s Disease. *n engl j med*, 13.
64. Weaver, F. M., Follett, K. A., Stern, M., Luo, P., Harris, C. L., Hur, K., et al. (2012). Randomized trial of deep brain stimulation for Parkinson disease: Thirty-six-month outcomes. *Neurology* 79, 55–65. doi:[10.1212/WNL.0b013e31825dcdc1](https://doi.org/10.1212/WNL.0b013e31825dcdc1).
65. Weintraub, D., Duda, J. E., Carlson, K., Luo, P., Sagher, O., Stern, M., et al. (2013). Suicide ideation and behaviours after STN and GPi DBS surgery for Parkinson’s disease: results from a randomised, controlled trial. *J Neurol Neurosurg Psychiatry* 84, 1113–1118. doi:[10.1136/jnnp-2012-304396](https://doi.org/10.1136/jnnp-2012-304396).
66. Weiss, D., Wächter, T., Meisner, C., Fritz, M., Gharabaghi, A., Plewnia, C., et al. (2011). Combined STN/SNr-DBS for the treatment of refractory gait disturbances in Parkinson’s disease: study protocol for a randomized controlled trial. *Trials* 12, 222. doi:[10.1186/1745-6215-12-222](https://doi.org/10.1186/1745-6215-12-222).
67. Welter, M.-L., Houeto, J.-L., Thobois, S., Bataille, B., Guenot, M., Worbe, Y., et al. (2017). Anterior pallidal deep brain stimulation for Tourette’s syndrome: a randomised, double-blind, controlled trial. *Lancet Neurol.* 16, 610–619. doi:[10.1016/S1474-4422(17)30160-6](https://doi.org/10.1016/S1474-4422(17)30160-6).
68. Welter, M.-L., Mallet, L., Houeto, J.-L., Karachi, C., Czernecki, V., Cornu, P., et al. (2008). Internal Pallidal and Thalamic Stimulation in Patients With Tourette Syndrome. *Arch. Neurol.* 65, 952-957. doi:[10.1001/archneur.65.7.952](https://doi.org/10.1001/archneur.65.7.952).
69. Wharen, R. E., Okun, M. S., Guthrie, B. L., Uitti, R. J., Larson, P., Foote, K., et al. (2017). Thalamic DBS with a constant-current device in essential tremor: A controlled clinical trial. *Parkinsonism & Related Disorders* 40, 18–26. doi:[10.1016/j.parkreldis.2017.03.017](https://doi.org/10.1016/j.parkreldis.2017.03.017).
70. Wojtecki, L., Groiss, S. J., Ferrea, S., Elben, S., Hartmann, C. J., Dunnett, S. B., et al. (2015). A Prospective Pilot Trial for Pallidal Deep Brain Stimulation in Huntington’s Disease. *Front. Neurol.* 6:177. doi:[10.3389/fneur.2015.00177](https://doi.org/10.3389/fneur.2015.00177).
71. Xie, T., Bloom, L., Padmanaban, M., Bertacchi, B., Kang, W., MacCracken, E., et al. (2018). Long-term effect of low frequency stimulation of STN on dysphagia, freezing of gait and other motor symptoms in PD. *J Neurol Neurosurg Psychiatry* 89, 989–994. doi:[10.1136/jnnp-2018-318060](https://doi.org/10.1136/jnnp-2018-318060).
